# Metabolic Syndrome and Its Associated Factors Among Patients With Schizophrenia Treated With Second-Generation Antipsychotics at Amanuel Mental Specialized Hospital, Ethiopia

**DOI:** 10.7759/cureus.69065

**Published:** 2024-09-10

**Authors:** Melak Gedamu Beyene, Solomon Teferra, Teferi G Fenta

**Affiliations:** 1 Department of Pharmacy, Addis Ababa University, Addis Ababa, ETH; 2 Department of Medicine, Addis Ababa University, Addis Ababa, ETH

**Keywords:** associated factors, ethiopia, metabolic syndrome, schizophrenia, second-generation antipsychotics

## Abstract

Background

Metabolic syndrome (MetS) encompasses a group of risk factors that increase the likelihood of developing cardiovascular diseases, thereby increasing the mortality rate. Second-generation antipsychotics (SGAs) are known for these side effects. This study aimed to determine the prevalence of MetS and its associated factors at Amanuel Mental Specialized Hospital.

Methods

A cross-sectional study was conducted from October 3, 2022, to August 31, 2023. Fasting blood sugar and lipid analysis were performed using the Dimension® EXL™ 200 Integrated Chemistry System (Siemens Healthineers, Malvern, PA, USA). A diagnosis of MetS was established using the modified National Cholesterol Education Program Adult Treatment Panel III (NCEP ATP-III) criteria. IBM SPSS Statistics for Windows, Version 25 (Released 2017; IBM Corp., Armonk, NY, USA) was used for analysis. A binary logistic regression model was employed, and a p-value less than 0.05 was considered significant.

Results

A total of 271 participants were enrolled in the study. Most subjects were male (90%) and had a mean age of 34.2 years, with an SD of 10.5. Most participants (70.8%) had abnormal waist circumference, followed by lower high-density lipoprotein cholesterol (HDL-C) at 42.8%. The prevalence of MetS was 35.8%. Gender (being female) (adjusted odds ratio or AOR 3, 95% CI: 1.2-7.4; p = 0.02) and olanzapine use (AOR 2.2, 95% CI: 1.3-3.7; p = 0.005) were predictors of MetS.

Conclusions

MetS is highly prevalent in patients treated with SGAs. Being female and olanzapine use were predictors of MetS. Clinicians managing these patients should screen and monitor the metabolic components used to diagnose MetS.

## Introduction

In the past, Reaven introduced the concept of 'syndrome X,' later renamed metabolic syndrome (MetS), suggesting its central role in the development of atherosclerotic cardiovascular diseases (ASCVDs) and type 2 diabetes mellitus (DMT2), primarily due to resistance to insulin action in target tissues. Its primary elements include dyslipidemia, marked by elevated triglycerides (TG) and apolipoprotein B (apoB)-containing lipoproteins, and low levels of high-density lipoproteins (HDLs). Additionally, there is an increase in arterial blood pressure (BP) and a disruption of glucose homeostasis [1].

MetS elevates the likelihood of non-communicable diseases (NCDs) and amplifies the expenses associated with their management. Mortality due to NCDs is twice as high in low- or lower-middle-income countries (LMICs) compared to high-income countries [2].

Individuals with psychiatric disorders face an elevated risk of premature death, primarily attributed to cardiovascular diseases (CVDs). Globally, individuals with severe psychotic disorders, like schizophrenia, exhibit a higher occurrence of MetS compared to the general population [3]. A meta-analysis report showed that the overall prevalence of MetS in psychiatric patients was 32.5%, and older age and duration of illness were reported as predictors of MetS [4]. Another finding also showed that 40% of schizophrenic patients will develop MetS [5].

Second-generation antipsychotics (SGAs) constitute a diverse group of medications. Among the presently available SGAs - namely, clozapine, aripiprazole, olanzapine, quetiapine, zotepine, risperidone, and ziprasidone - each agent possesses distinct pharmacological characteristics and a unique binding profile to multiple receptors. These variations contribute to differences in side effects [6]. Use SGAs with caution, as they can contribute to weight gain and pose risks of obesity, dyslipidemia, diabetes, accelerated CVD, and even premature mortality. Although the exact mechanism is not fully understood, it is believed to be associated with heightened appetite and subsequent weight gain, progressing to obesity, insulin resistance, and dyslipidemia [7].

One meta-analysis has indicated that the utilization of SGAs is linked to the highest risk of MetS compared to first-generation antipsychotics (FGAs), with clozapine and olanzapine being identified as major contributors [8]. MetS in schizophrenia patients is also influenced by various factors, including demographic, clinical, and lifestyle elements: an unhealthy lifestyle, decreased physical activity, smoking, a poor diet, and genetic predisposition [9].

The current clinical practice in schizophrenia management in Ethiopia lacks continuous monitoring of MetS in patients treated with SGAs. These antipsychotics, while effective for managing schizophrenia, are associated with an increased risk of MetS, which includes conditions such as obesity, hypertension, dyslipidemia, and insulin resistance. Despite this known risk, routine screening and ongoing assessment for MetS are not consistently integrated into clinical care. This oversight can lead to delayed diagnosis and management of MetS, exacerbating long-term health outcomes for these patients. Incorporating regular monitoring of metabolic indicators into standard care is crucial to mitigate these risks and improve overall patient health. There is a lack of data regarding the prevalence of MetS and its predictors in individuals with schizophrenia who are prescribed SGAs in Ethiopia. Addressing this gap is of utmost importance in understanding the prevalence of MetS and its determinants among individuals with schizophrenia who are being treated with SGAs. Hence, this study aimed to determine the prevalence of MetS and its associated factors in patients treated with SGAs.

## Materials and methods

Study setting and period

This research was carried out at Amanuel Mental Specialized Hospital (AMSH), Addis Ababa, Ethiopia. AMSH, established in 1930, has provided mental health treatment and rehabilitation since then. The hospital has 300 beds - 277 dedicated to inpatient care and 23 for emergency cases. In addition to inpatient services, it offers outpatient care, with an estimated 115,000 outpatient visits annually. Furthermore, AMSH serves as a referral hospital for psychiatric cases from all regions across the country and provides specialized training in psychiatry. This study was conducted at AMSH from October 3, 2022, to August 31, 2023.

Sample size determination and sampling technique

The sample size was calculated using a single population proportion formula, with a 95% confidence interval and a 5% margin of error. We considered various prevalence rates of MetS and other variables to determine the sample size. Therefore, using a prevalence rate of 21.5% reported from a previous study at AMSH resulted in the maximum required sample size [10]. An additional 10% was included to account for contingencies, bringing the total to 287 participants for data collection. The study participants were selected using a simple random sampling method, with numbers generated using Excel (Microsoft® Corp., Redmond, WA, USA). 

Study design and participants

An institution-based cross-sectional study design was employed. The source population included all individuals receiving care at AMSH and participating in the Neuropsychiatric Genetics in African Populations (NeuroGAP-Psychosis) project. Data from this project were used to identify patients with confirmed schizophrenia. The study population specifically comprised patients with schizophrenia who had been on risperidone or olanzapine for at least three months, as these medications are known to predispose individuals to MetS, and who met the inclusion criteria during the study period.

Inclusion and exclusion criteria

The inclusion criteria comprised the absence of physical complications or other psychiatric disorders; no signs of resistance to antipsychotic treatment; adherence to the Diagnostic and Statistical Manual of Mental Disorders (DSM-5) criteria for schizophrenia; and limited concurrent medication use to benzodiazepines for anxiety or agitation, and anticholinergics for extrapyramidal side effects. Participants also needed to be 18 years of age or older, willing to participate voluntarily, and not in an acute psychotic episode. The exclusion criteria included a diagnosis of severe, unstable medical or neurological conditions; the presence of an intellectual disability; and severe cognitive decline that would hinder understanding of the study questions.

Study variables

The dependent variable was the prevalence of MetS. The independent variables comprised socio-demographic and clinical characteristics, such as age, gender, marital status, family income level, employment status, medications prescribed, comorbidity, duration of the disease, time of treatment initiation, dose of the medication, duration of treatment, social drug use, lipid profiles, fasting blood sugar (FBS), and positive and negative syndrome scale (PANSS) score [11].

Data collection process

The data administration office of the NeuroGAP project granted us access to a comprehensive database containing detailed information on patients diagnosed with schizophrenia who were currently undergoing treatment with olanzapine or risperidone. After obtaining the identification numbers of these patients, their phone numbers were retrieved for direct communication. Patients who expressed consent to participate had their medical charts retrieved from the hospital's card office. During the initial contact, the study's objectives were communicated, and patients willing to participate were invited to the hospital, where their medical charts were retrieved from the archives.

The researchers designed questionnaires to collect socio-demographic information from the study participants. These questionnaires were intended to gather details about the patients' backgrounds, such as age, gender, education, occupation, and other socio-demographic factors that could be related to MetS. Additionally, a data abstraction format was prepared to extract clinical information, including details about the specific medications prescribed, their dosages, and any pertinent clinical data that could influence treatment outcomes. Data collection was carried out by two trained psychiatry professionals with MSc degrees.

Ensuring data quality was of the utmost importance throughout the study. Various measures were implemented to maintain the accuracy and integrity of the data. The clarity and comprehensibility of the questionnaire were closely scrutinized, with necessary amendments made to enhance its quality. Training was provided to ensure that data collectors were well-prepared for accurate data collection. Continuous supervision by the principal investigator was upheld to address potential challenges and maintain data quality. Rigorous checks for completeness were conducted on the collected data.

Biochemical Measurements

A volume of 3-4 mL of whole blood samples was collected in the morning from the antecubital vein using vacutainer tubes without anticoagulant, following an 8-12-hour fasting period, by a skilled laboratory technologist for lipid and FBS analyses. The blood was allowed to clot for 30 minutes at room temperature, and the serum was obtained by centrifugation (3,000 rpm for 10 minutes). The serum was analyzed at the AMSH central clinical chemistry laboratory on the same day. Serum TG was measured using a glycerol oxidase enzymatic method, HDL-cholesterol (HDL-C) by a cholesterol oxidase enzymatic method, and FBG was measured using an enzymatic (glucose oxidase) colorimetric method. All determinations were performed with a fully automated clinical chemistry analyzer (Dimension® EXL™ 200 Integrated Chemistry System; Siemens Healthineers, Malvern, PA, USA). The same manufacturer-supplied reagent was used since it is a closed system. Determination of the concentration of FBS, TG, and HDL was done using a bichromatic (340 and 383 nm), (510 and 700 nm), and (600 and 700 nm) endpoint technique, respectively. Quality assurance was maintained by analyzing two levels of quality control (QC) materials with known concentrations for each parameter. For FBG measurement, QC materials with known glucose concentrations were analyzed at least once each day of use. Similarly, for TG and HDL-C tests, two levels of QC materials with known triglyceride and HDL-C concentrations were also analyzed each day the assay was performed, respectively.

Anthropometric Measurements

BP: Three BP measurements were taken by standard mercury sphygmomanometers after the study participants were seated for five minutes. To the nearest 1 mmHg, readings were taken from seated participants with the right arm resting and the palm facing upward. Two readings were taken five minutes apart, and the average was then calculated to determine the final result. If the difference between the first and the second readings was ≥10 mmHg for systolic blood pressure (SBP) and ≥6 mmHg for diastolic blood pressure (DBP), then a third measurement was made, and the mean of all three measurements was taken [12].

Height and weight measurements: Patients' weight (kg) and height (cm) were measured following the WHO stepwise approach, utilizing a digital electronic adult scale with both weight and height measurement features. Body weight was recorded to the nearest 0.05 kg, and height was measured to the nearest 0.5 cm, with the subject's head on the Frankfurt plane. Height and weight were measured twice, and the average of each variable was used for the final record. Training was provided to the data collectors on how to perform regular calibration of the equipment to ensure the accuracy and reliability of the readings, thereby maintaining the overall quality of the measurements.

Body mass index (BMI): BMI is calculated by dividing weight in kilograms by the square of height in meters. This method provides a simple way to assess body fatness and is widely employed in clinical settings. BMI classifications are as follows: underweight BMI: <18.5, average weight BMI: 18.5 to 24.9, overweight BMI: 25.0 to 29.9, and obese BMI: ≥30.

A diagnosis of MetS in the present study was established using the modified National Cholesterol Education Program Adult Treatment Panel III (NCEP ATP-III) criteria, which require the presence of three out of the following five abnormalities: high waist circumference (WC) (≥80 cm for women and ≥94 cm for men of sub-Saharan African origin); SBP ≥130 and/or DBP ≥85 mm Hg (or on treatment for hypertension); TG levels ≥150 mg/dL (or on specific treatment for this abnormality); HDL-C <40 mg/dL for men and <50 mg/dL for women (or on specific treatment for this abnormality); and FBS ≥100 mg/dL (or on treatment for diabetes mellitus) [13].

Statistical analysis

The data underwent a rigorous processing phase to ensure its reliability and quality. This involved comprehensive cleaning to address completeness issues, followed by coding, entry, and subsequent analysis utilizing IBM SPSS Statistics for Windows, Version 25 (Released 2017; IBM Corp., Armonk, NY, USA). Categorical variables were described as counts and percentages and compared between groups using Pearson’s Chi-square test or Fisher’s exact test. Continuous variables were checked for normality using the Shapiro-Wilk test. Normally distributed variables were presented as mean and standard deviation, while non-normally distributed variables were presented as median and interquartile range (IQR). Continuous variables were compared between groups (those with and without MetS) using the independent sample t-test when normally distributed and the two-sample Wilcoxon rank sum (Mann-Whitney U) test when not normally distributed. Binary logistic regression analysis was employed to identify predictors of MetS in patients with schizophrenia. Candidate variables for the multivariable binary logistic regression analysis were selected based on a p-value less than 0.25 from the univariate analysis. Independent variables with a p-value less than 0.05 were considered significantly associated with the outcome variable, and these associations were reported using adjusted odds ratios (AORs) accompanied by 95% confidence intervals. The parameters employed for classifying MetS were excluded from the binary logistic regression for predictor identification due to their inherent relationship with the outcome variable.

## Results

Patient characteristics

The socio-demographic characteristics of subjects with and without MetS included in the study are presented in Table 1. A total of 271 participants were included in the analysis. Most of the study participants were male (244, or 90%). A significant difference was observed between males and females in relation to MetS (p = 0.01). The mean age of the study participants was 34.2 years, with a standard deviation of 10.5 years. Subjects with MetS had a slightly higher mean age. Most study participants had an education level below college (201, or 74.2%) and were urban dwellers (213, or 78.6%). Employment status showed a slight majority of 153 participants (56.5%) were unemployed. The majority of participants (249, or 91.9%) lived with their families. Almost half of the study subjects (127, or 46.8%) reported engaging in substance use.

**Table 1:** Socio-demographic characteristics of patients with schizophrenia, Ethiopia (N = 271). *CBHI and free; **Catholic, no religion CBHI: Community-based health insurance; IQR: Interquartile range; MetS: Metabolic syndrome

Variables	MetS			
	Without MetS, N (%)	With MetS, N (%)	Total, N (%)	p-value
Age (years) (median, IQR)	32 (14.0)	36 (12.0)	32 (14.0)	0.26
Age category				
≤45	152 (64.4)	84 (35.6)	236 (87.1)	0.86
>45	22 (62.9)	13 (37.1)	35 (12.9)	
Gender				
Male	163 (66.8)	81 (33.2)	244 (90.0)	0.01
Female	11 (40.7)	16 (59.3)	27 (10.0)	
Educational background				
Below college	128 (63.7)	73 (36.3)	201 (74.2)	0.76
College and above	46 (65.7)	24 (34.3)	70 (25.8)	
Place of residence				
Urban	134 (62.9)	79 (37.1)	213 (78.6)	0.39
Rural	40 (69.0)	18 (31.0)	58 (21.4)	
Marital status				
Single	114 (65.9)	59 (34.1)	173 (63.8)	0.71
Married	60 (61.2)	38 (38.8)	98 (36.2)	
Health service fee				
Paying out-of-pocket	26 (58.8)	19 (42.2)	45 (16.6)	0.33
Other means*	148 (65.5)	78 (34.5)	226 (83.4)	
Employment status				
Employed	70 (59.3)	48 (40.7)	118 (43.5)	0.14
Unemployed	104 (68.0)	49 (32.0)	153 (56.5)	
Living arrangement				
Living alone	11 (50.0)	11 (50.0)	22 (8.1)	0.18
Living with others	163 (65.5)	86 (34.5)	249 (91.9)	
Religious affiliation				
Orthodox Christian	78 (60.9)	50 (39.1)	128 (47.2)	0.49
Muslim	67 (64.4)	37 (35.6)	104 (38.4)	
Protestant	25 (73.5)	9 (26.5)	34 (12.6)	
Others**	4 (80.0)	1 (20.0)	5 (1.8)	
Substance use				
Yes	76 (59.8)	51 (40.2)	127 (46.8)	0.16
No	98 (68.1)	46 (31.9)	144 (53.2)	

Biochemical and anthropometric measurements

The major findings from the physical and laboratory measurements of subjects with schizophrenia revealed significant insights into their health status. The majority of 179 (66.1%) study participants had average weight. Abdominal obesity, as indicated by WC, was prevalent in 192 (70.8%) of the study subjects. About 45 (16.6%) of the subjects had high SBP, and about 41 (15.1%) had high DBP. However, a notable proportion, 60 (22.1%), exhibited higher overall BP. About one-third, 79 (29.2%), had high FBS, while 97 (35.8%) had elevated TG. Furthermore, about 116 (42.8%) study participants had low levels of HDL (Table 2).

**Table 2:** Physical and laboratory measurements of patients with schizophrenia, Ethiopia (N = 271). BMI: Body mass index; DBP: Diastolic blood pressure; SBP: Systolic blood pressure; FBS: Fasting blood sugar; HDL: High-density lipoprotein; WC: Waist circumference; TG: Triglycerides

Variables	Frequency (%)
BMI (kg/m^2^)	
Underweight (<18.5)	48 (17.6)
Average weight (18.5-24.9)	179 (66.1)
Overweight (25.0-29.9)	33 (12.2)
Obese (≥30)	11 (4.1)
WC (cm)	
Normal WC	79 (29.2)
Abnormal WC	192 (70.8)
SBP (mmHg)	
Normal SBP	226 (83.4)
High SBP	45 (16.6)
DBP (mmHg)	
Normal DBP	230 (84.9)
High DBP	41 (15.1)
BP (mmHg)	
Normal BP (mmHg)	221 (77.9)
High BP (mmHg)	60 (22.1)
FBS (mg/dL)	
Normal FBS	192 (70.8)
High FBS	79 (29.2)
TG (mg/dL)	
Normal TG	174 (64.2)
High TG	97 (35.8)
HDL (mg/dL)	
Normal HDL	155 (57.2)
Low HDL	116 (42.8)

Outcome measures

In the present study, the investigation into the prevalence of MetS in patients with schizophrenia in Ethiopia revealed a high prevalence of MetS. The data unveiled that 97 (35.8%) of the study subjects exhibited MetS. The comprehensive analysis of clinical and metabolic biomarkers revealed several notable findings. The findings showed that there is a variation in the median and IQR of the two groups of patients with MetS and without MetS in relation to WC, BMI, SBP, DBP, FBG, TG, duration of treatment, chlorpromazine (CPZ) equivalent doses, and PANSS (Table 3).

**Table 3:** Clinical and metabolic biomarkers characteristics of patients with schizophrenia, Ethiopia (N = 271). BMI: Body mass index; BP: Blood pressure; cm: Centimeter; DBP: Diastolic blood pressure; dL: Deciliter; DUP: Duration of untreated psychosis; F: Female; FBG: Fasting blood glucose; HDL-C: High-density lipoprotein cholesterol; IQR: Interquartile range; kg: Kilogram; M: Male; m: Meter; mg: Milligram; mmHg: Millimeter mercury; PANNS: Positive and negative syndrome scale; SBP; Systolic blood pressure; SD: Standard deviation; TG: Triglyceride; WC: Waist circumference

Variables	MetS			
	Without MetS (median, IQR)	With MetS (median, IQR)	Total (median, IQR)	p-value
Duration of illness (years)	5 (7.0)	6 (11.0)	6 (8.0)	0.59
Duration of treatment (years)	4 (7.8)	6 (9.0)	5 (8.0)	0.14
CPZ equivalent dose (mg)	200 (100.0)	300 (200.0)	200 (100.0)	0.02
WC (cm)	98 (11.0)	102 (4.0)	99 (12.0)	<0.001
BMI (kg/m^2^)	21.2 (3.5)	21.8 (7.0)	21.3 (4.0)	0.11
SBP (mmHg)	110 (20.0)	120 (20.0)	110 (10.0)	<0.001
DBP (mmHg)	72.5 (10.0)	80 (20.0)	78 (10.0)	0.01
FBG (mg/dL)	83 (18.0)	104 (17.0)	87 (14.0)	<0.001
TG (mg/dL)	120 (73.8)	168 (138.0)	126 (93.0)	0.01
HDL-C (mg/dL)	44 (15.0)	35 (10.0)	43 (16.0)	<0.001
PANSS total	62.5 (46.3)	62 (66.0)	62 (51.0)	0.81
DUP (years) (mean, SD)	1.1 (2.5)	0.97 (1.5)	0.9 (2.2)	0.07
	N (%)	N (%)	N (%)	
Abnormal WC (≥94 M, ≥80 F)	101 (52.6)	91 (47.4)	192 (70.8)	<0.001
SBP ≥ 130 mmHg	9 (20.0)	36 (80.0)	45 (16.6)	<0.001
DBP ≥ 85 mmHg	10 (24.4)	31 (75.6)	41 (15.3)	<0.001
Medication type				
Risperidone	112 (72.3)	43 (27.7)	155 (57.2)	<0.001
Olanzapine	62 (53.4)	54 (46.6)	116 (42.8)	
Medication switch				
Yes	88 (59.5)	60 (40.5)	148 (54.6)	0.07
No	86 (69.9)	37 (30.1)	123 (45.4)	
Abnormal BP ≥130/≥85 mmHg	16 (26.7)	44 (73.3)	60 (22.1)	-
Lower HDL	43 (37.1)	73 (62.9)	116 (42.8)	<0.001
Higher TG (≥150 mg/dL)	37 (38.1)	60 (61.9)	97 (35.8)	<0.001
FBG ≥ 100 mg/dL	26 (32.9)	53 (61.9)	79 (29.2)	<0.001

**Figure 1:**
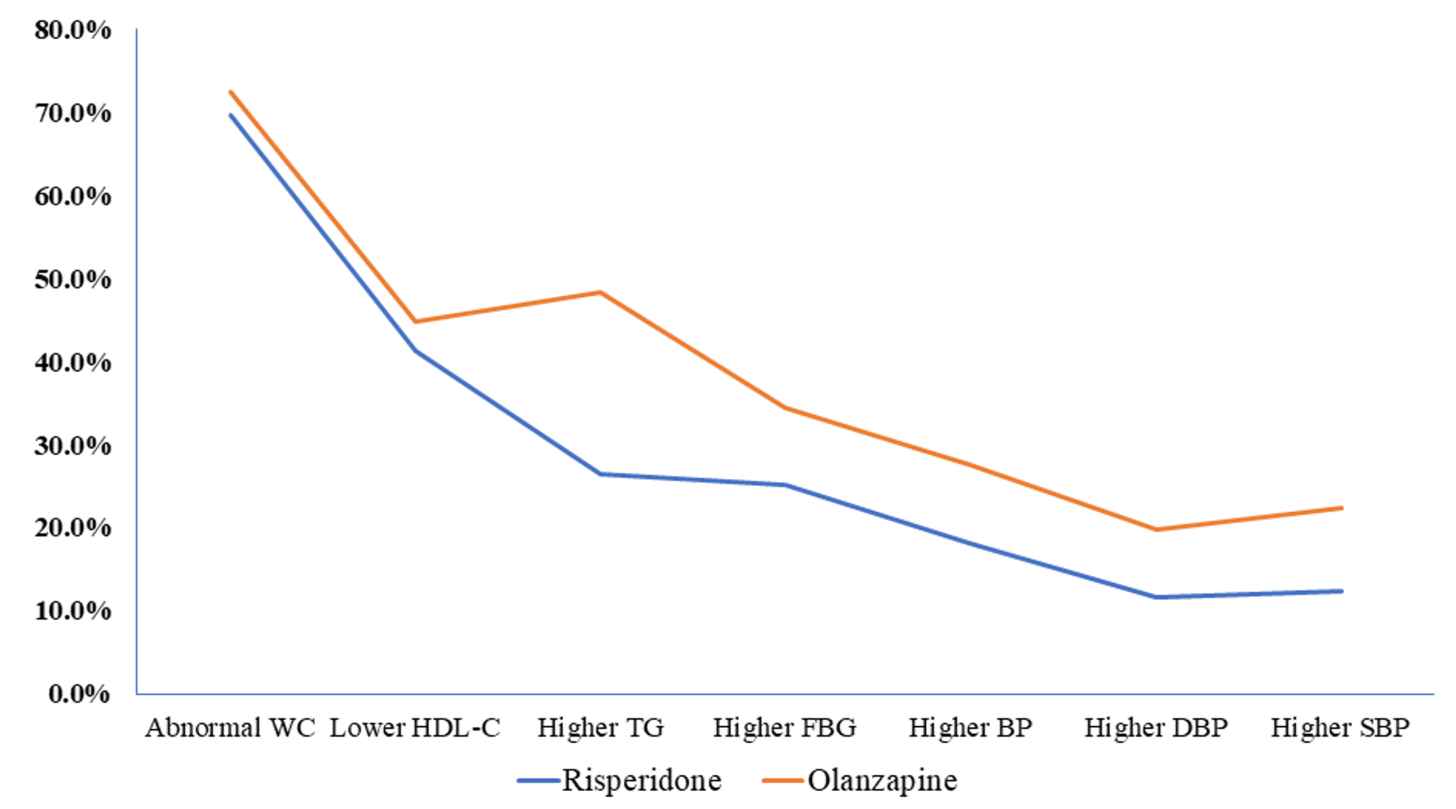
Metabolic biomarker abnormalities in patients with schizophrenia treated with atypical antipsychotics, Ethiopia (N = 271). WC: Waist circumference; HDL-C: High-density lipoprotein cholesterol; TG: Triglyceride; FBG: Fasting blood glucose; BP: Blood pressure; DBP: Diastolic blood pressure; SBP: Systolic blood pressure

The study also compared the prevalence of metabolic biomarker abnormalities between medications. The results showed higher levels of metabolic biomarker abnormalities in patients receiving olanzapine, compared to those on risperidone (Figure 1).

**Figure 2:**
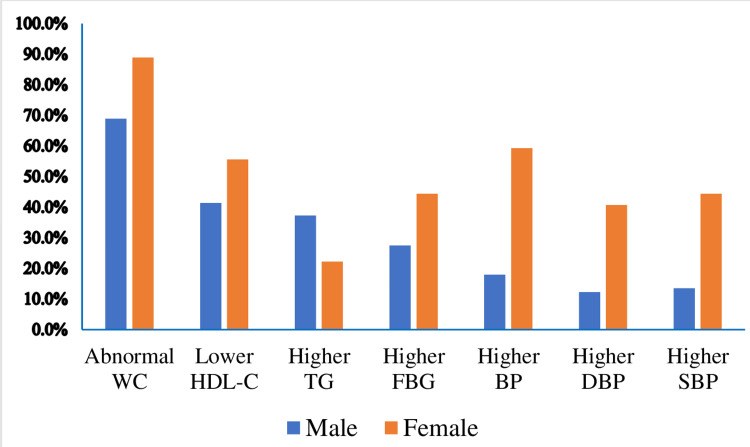
Metabolic biomarker abnormalities in gender differences in patients with schizophrenia, Ethiopia (N = 271). WC: Waist circumference; HDL-C: High-density lipoprotein cholesterol; TG: Triglyceride; FBG: Fasting blood glucose; BP: Blood pressure; DBP: Diastolic blood pressure; SBP: Systolic blood pressure

This study assesses the prevalence of MetS in patients who have been on risperidone and olanzapine treatment. The results indicate a higher prevalence of MetS in the olanzapine group (54, or 46.6%) compared to the risperidone group (43, or 27.7%). The report also revealed a higher prevalence of MetS in the female group (16, or 59.3%). This investigation examined the prevalence of metabolic biomarker abnormalities among male and female study participants. The results revealed a higher incidence of metabolic biomarker abnormalities in female patients across all measurements, except for higher triglyceride levels observed in male participants (Figure 2).

Following a univariate logistic regression analysis, a multivariate binary logistic regression analysis was conducted to identify predictors of MetS. The results of the multivariate logistic regression revealed that gender (being female) and medication type (being on olanzapine) emerged as statistically significant predictors of MetS (AOR 3, 95% CI: 1.2-7.4; p = 0.02, and AOR 2.2, 95% CI: 1.3-3.7; p = 0.005, respectively). The likelihood of MetS occurrence was three times higher in the female group compared to male participants, and patients receiving olanzapine treatment had 2.2 times higher odds of developing MetS compared to those on risperidone treatment. However, variables such as employment status, substance use, PANSS total score, BMI, duration of untreated psychosis (DUP), and symptom remission did not show significant associations with the occurrence of MetS (Table 4).

**Table 4:** Multivariate binary logistic regression analysis of predictors of metabolic syndrome in patients with schizophrenia, Ethiopia (N = 271). *p < 0.05 AOR: Adjusted odds ratio; BMI: Body mass index; CPZ: Chlorpromazine; COR: Crude odds ratio; SD: Standard deviation; DUP: Duration of untreated psychosis; PANSS: Positive and negative syndrome scale

Variables	Without MetS	With MetS	COR (95% CI)	AOR (95% CI)
Gender				
Male	163 (66.8)	81 (33.2)	1	1
Female	11 (40.7)	16 (59.3)	2.9 (1.3, 2.6)	3 (1.2, 7.4)*
Employment status				
Employed	70 (59.3)	48 (40.7)	1	1
Unemployed	104 (68)	49 (32)	0.67 (0.4, 1.1)	0.7 (0.4, 1.1)
Substance use				
Yes	76 (59.8)	51 (40.2)	1	1
No	98 (68.1)	46 (31.9)	0.7 (0.4, 1.2)	0.6 (0.3, 1.0)
BMI				
<25	150 (66.1)	77 (33.9)	1	1
≥25	24 (54.5)	20 (45.5)	1.6 (0.8, 3.1)	1.4 (0.7, 2.9)
Medication type				
Risperidone	112 (72.3)	43 (27.7)	1	1
Olanzapine	62 (53.4)	54 (46.6)	2.3 (1.4, 3.8)	2.2 (1.3, 3.7)*
Medication switch				
Yes	88 (59.5)	60 (40.5)	1	1
No	86 (69.9)	37 (30.1)	0.6 (0.4, 1.1)	0.6 (0.4, 1.1)
PANSS total score (mean, SD)	68.6 (35.7)	75.5 (36)	1.01 (0.9, 1.0)	1 (0.9, 1.0)
DUP (years)				
<1	116 (61.1)	74 (38.9)	1	1
≥1	58 (71.6)	23 (28.4)	0.6 (0.4, 1.1)	0.6 (0.3, 1.1)

## Discussion

The present study investigated and determined the prevalence of MetS and its predictors in patients diagnosed with schizophrenia and undergoing treatment with SGAs (risperidone or olanzapine) for a minimum of three months. The diagnosis of MetS in this study adhered to the modified version of the NCEP ATP-III criteria [13].

The main finding indicated that the prevalence of MetS is high and is reported in 35.8% of patients. This report is in line with previous findings from Eastern Ethiopia (36.5%) [14], Hong Kong (35%) [15], and a global report from a systematic review and meta-analysis (25-50%) [16]. On the contrary, our finding is higher than reports from Thailand (22.8%) [17], Spain (26.5%) [18], Uganda (23.51%) [19], and South Africa (23.2%) [20]. This difference might be partly explained by the fact that different definitions and cutoffs for WC have been employed across different ethnic diversities [13]. However, our finding demonstrated a lower MetS prevalence than reports from the USA (49.2%) [21] and England (57%) [22]. This variation may be attributed to lifestyle differences, as these studies were conducted in high-income countries, contributing to variations in nutritional status and better compliance with their medications.

The metabolic abnormalities were investigated, and it was found that the majority of the subjects had abnormal WC (70.8%), followed by low HDL-C (42.8%). This finding aligns with previous studies conducted in India [23]. On the other hand, a previous study conducted in Ethiopia reported that the most common metabolic abnormality identified was low HDL-C [10]. This difference might be due to the difference in the cutoff points for WC, which might be overestimated in our measurement because we used the harmonized version, which accounts for ethnic differences [13].

In the current study, we found a significant gender difference in the prevalence of MetS in the sampled population (female vs. male: 59.3% vs. 33.2%). Differences in physiology, level of physical activity, and psychological factors between females and males could partly explain the disparity in the prevalence of MetS between genders. Similarly, all metabolic abnormality biomarkers were found to have a higher prevalence in female patients across all measurements, except for higher TG, which was observed in male participants. This finding aligns with other reports that women have a lower concentration of TG in plasma than men [24]. These sex differences in total fasting plasma TG concentrations are attributed entirely to lower very low-density lipoprotein (VLDL)-TG concentrations in females [25].

The likelihood of MetS occurrence was three times higher in the female participants compared to male participants, with statistical significance (AOR 3, 95% CI: 1.2, 7.4; p = 0.02). Similar to this finding, a previous report in Ethiopia found that more females than males (17.1% of males and 29.6% of females) [10] and in Taiwan (38.9% of females and 31.5% of males) were diagnosed as having MetS [26]. This higher prevalence in the female group may be attributed to hormonal differences between male and female subjects, with accumulated data persuasively demonstrating that significant heterogeneity exists between men and women developing MetS at large, which could be explained by differences in hormonal regulation of body fat distribution [27].

This study also showed a statistically significant difference in the prevalence of MetS concerning medication type (olanzapine vs. risperidone: 46.6% vs. 27.7%). All the parameters used for measuring metabolic abnormalities were higher in patients prescribed olanzapine than in those prescribed risperidone. Patients receiving olanzapine treatment had 2.2 times higher odds of developing MetS compared to those on risperidone treatment (AOR 2.2, 95% CI: 1.3, 3.7; p = 0.005), and the associations are statistically significant. Evidence shows that SGAs differ in their propensity to cause MetS, with the highest association found in patients taking olanzapine [28]. A clinical trial from the Clinical Antipsychotic Trials of Intervention Effectiveness (CATIE) determined the prevalence of MetS in patients with schizophrenia and found a significant increase in the incidence of MetS in the olanzapine group when compared to the risperidone group [29].

Even though a longer duration of illness has been reported to be associated with a higher prevalence of MetS in one study [23], we found no strong association with the occurrence of MetS in the present study. This difference can be partly explained by differences in disease severity and genetic variations among the study participants [30]. Variables such as employment status, substance use, PANSS total score, BMI, DUP, and symptom remission did not show significant associations with the occurrence of MetS in the present study. However, a previous report in Ethiopia showed that the PANSS total score was strongly associated with MetS and became a predictor of the presence of MetS [10]. This difference may be explained by the patient's disease condition or severity.

Strengths and limitations

The present study has some limitations worth mentioning. First, this study is a single-center cross-sectional study; therefore, the generalizability of the study could be improved, and the causation of MetS and its predictors cannot be ascertained. Secondly, there was a gender imbalance in our included sample, meaning that the sample size of females was significantly lower than that of males. Third, the relevant factors for MetS included in this study might be limited, and there may be other relevant contributing factors that we should have included in the analysis. For example, we did not assess the participants' dietary habits, physical activities, and genetic variability, yet these are essential determinants of MetS. This study has strengths, as it employed the most updated clinical guideline, called the harmonized criteria, which accounts for racial differences to define MetS, and we conducted the laboratory analyses in a quality-accredited center.

## Conclusions

In conclusion, the present study highlighted that MetS is highly prevalent in patients with schizophrenia treated with SGAs. Being female and on olanzapine treatment were predictors of the occurrence of MetS. Recognizing and effectively addressing the needs of this at-risk population holds significant importance for comprehensive clinical care. In addition, antipsychotic medication selection should be done cautiously. Further studies are also recommended to determine the temporal relationship between SGAs and MetS in the Ethiopian population.
